# Sleep, Mental Health, and the Need for Physical and Real-Life Social Contact with (Non-)Family Members during the COVID-19 Pandemic: A Bayesian Network Analysis

**DOI:** 10.3390/jcm13133954

**Published:** 2024-07-05

**Authors:** Aurore Roland, Louise Staring, Martine Van Puyvelde, Francis McGlone, Olivier Mairesse

**Affiliations:** 1 Brain, Body and Cognition, Department of Psychology, Faculty of Psychology and Educational Sciences, Vrije Universiteit Brussel, 1050 Brussels, Belgium; 2 Brussels University Consultation Center, Department of Psychology, Faculty of Psychology and Educational Sciences, Vrije Universiteit Brussel, 1050 Brussels, Belgium; 3 Vital Signs and PERformance Monitoring (VIPER), LIFE Department, Royal Military Academy, 1000 Brussels, Belgium; 4 Clinical and Lifespan Psychology, Department of Psychology, Faculty of Psychology and Educational Sciences, Vrije Universiteit Brussel, 1050 Brussels, Belgium; 5 School of Natural Sciences & Psychology, Faculty of Science, Liverpool John Moores University, Liverpool L3 3AF, UK; 6 Department of Neuroscience and Biomedical Engineering, School of Science, Aalto University, 00076 Espoo, Finland; 7 Laboratoire de Psychologie Médicale et Addictologie, CHU/UVC Brugmann, 1020 Brussels, Belgium

**Keywords:** sleep, mental health, touch, pandemic, lockdown, long COVID, social isolation

## Abstract

**Background/Objectives**: The forced social isolation implemented to prevent the spread of the COVID-19 virus was accompanied by a worsening of mental health, an increase in insomnia symptoms, and the emergence of ‘skin hunger’—an increased longing for personal touch. This study aimed to enhance our understanding of the interconnection between sleep, mental health, and the need for physical (NPC) and real-life social contact (NRL-SC). **Methods**: A total of 2827 adults participated in an online survey during the second COVID-19 lockdown. A Bayesian Gaussian copula graphical model (BGCGM) and a Bayesian-directed acyclic graph (DAG) were estimated, and mixed ANOVAs were carried out. **Results**: NPC with non-family members (*t*(2091) = 12.55, *p* < 0.001, *d* = 0.27) and relational lifestyle satisfaction (*t*(2089) = 13.62, *p* < 0.001, *d* = 0.30) were lower during the second lockdown than before the pandemic. In our BGCGM, there were weak positive edges between the need for PC and RL-SC on one hand and sleep and mental health on the other. **Conclusions**: During the second lockdown, people craved less physical contact with non-family members and were less satisfied with their relational lifestyle than before the pandemic. Individuals with a greater need for PC and RL-SC reported poorer mental health (i.e., worry, depression, and mental fatigue).

## 1. Introduction

The year 2020 was marked by the COVID-19 pandemic, which led governments to implement measures to curb its spread. In Belgium, the first lockdown commenced on March 10th, and social distancing measures were implemented such that individuals could interact with only one person with whom they did not reside. This was followed a few days later by closing schools and non-essential stores, prohibiting travel, and instating telework. In May and June, many measures were lifted, such as people were permitted to return to work, stores reopened, and travel was once again allowed. Social measures were lifted until July when everyone was allowed to see up to fifteen people per week. However, a few weeks later, social measures became stricter again, and eventually, on the 2nd of November, individuals were restricted to inviting only one person to their homes with whom they could have close contact. Those living alone could be in close contact with two people. Teleworking and remote learning were reinstated, non-essential stores had to close, and wearing a mask when leaving the house became mandatory [1]. Thus, a second lockdown was then introduced. Hale et al. [2] developed a stringency index, ranging from 0 to 100, indicating the strictness of the measures taken by a country’s government. During the first lockdown, Belgium soared to a stringency index of 81.48, and during the second lockdown, the stringency index peaked at 75.93.

This forced social isolation was accompanied by a worsening of mental health and an increase in insomnia symptoms [3,4,5]. Additionally, it gave rise to the phenomenon called “skin hunger” [6], characterized by an increased longing for interpersonal touch [7]. The latter has been associated with a lower experienced quality of life [8], increased mental health symptoms [9], and sleep disturbances [10] during the pandemic. One’s living situation has been related to the need for interpersonal physical contact, with those living alone reporting higher levels of touch deprivation than those cohabiting during the COVID-19 pandemic [7,10].

This study is one of a few that took place during the second COVID-19-related lockdown and, as far as we know, the first regarding touch and social contact. We aimed to enhance understanding of the interconnection between sleep, mental health, and the need for interpersonal physical (NPC) and real-life social contact (NRL-SC) with family and non-family members during the second COVID-19 lockdown (starting 2 November 2020) using Bayesian network analysis, a recent and innovative method.

Our first research question was to explore the relationships between NPC and NRL-SC with family and non-family members and mental health and sleep complaints during the second COVID-19 lockdown. We expected people with greater NPC and NRL-SC to have worse mental health and more sleep complaints. Our second research question was: what were the roles of age, sex, cohabitation, and satisfaction with relational lifestyle play in this relationship? We expected women to report more sleep complaints, worrying, and depressive feelings and for relational lifestyle satisfaction to be negatively related to mental health and sleep complaints. Finally, the third research question was: were NPC and NRL-SC with family and non-family members and relational lifestyle satisfaction different during the second lockdown than before the COVID-19 pandemic? We expected NPC with family members to increase and NPC with non-family members to decrease during the lockdown. This final research question had two subquestions: we wished to know whether there was an interaction effect for people living alone with (not) being in a romantic relationship. For the people cohabiting, we wished to know whether there was an interaction effect with whom they were cohabiting.

## 2. Materials and Methods

### 2.1. Participants

The participants of this study included 2827 adults with a mean age of 41.42 years (*SD* = 14.31 years). The minors who replied to the questionnaire were excluded from the analysis. Males (*M* = 43.87 years; *SD* = 14.55) were significantly older than females (*M* = 40.13 years, *SD* = 14.13) (*t*(2825) = 6.61, *p* ≤ 0.001, *d* = 0.27). Most participants in this study were female (*n* = 1923, 68.02%). See Table 1 for a breakdown of who the participants cohabitated with based on their age categories. This information was not provided by 67 participants. Of the people living alone, 360 were single, and 144 were in a relationship. Thirty-three participants did not provide this information.

### 2.2. Materials and Procedure

This study was approved by the Comité d’éthique hospitalier (hospital ethical committee) of the Brugmann Hospital (Brussels, Belgium; number: CE 2023/40). Informed consent of the participants was obtained. The survey was launched and sent out on 23 November 2020 in French and Dutch via hospital newsletters, regular media (newspapers and radio), and social media. Data were retrieved on 6 February 2021. Most respondents replied to the Dutch version (75.6%).

The survey consisted of 74 questions, of which only those pertinent to this study were reported. First, biographical data such as sex, age, and cohabitation status were collected. Additional details about their cohabitants were requested for those indicating cohabitation. Then, a set of questions were asked twice: once regarding their situation during the pre-pandemic period and once regarding the second lockdown of COVID-19 (winter 2020–2021). These questions included the Insomnia Severity Index (ISI). The ISI is a seven-item scale measuring the severity of difficulties initiating sleep (DIS), maintaining sleep (DMS), early morning awakenings (EMA), sleep dissatisfaction, interference of sleep difficulties with daytime functioning, noticeability of sleep problems by others and distress caused by the sleep difficulties. These items are answered on a five-point Likert scale (no problem to very severe problem) [11]. It is a reliable and validated questionnaire for the measurement of insomnia [12]. In our analysis, only the first four of the seven items were used to avoid having too many sleep-related items in the analyses. In addition to insomnia-related inquiries, information on participants’ satisfaction with their relational lifestyle (married, single, cohabiting, etc.) was assessed. We also explored the need for interpersonal physical contact (NPC) and real-life social contact (NRL-SC) with both family (including romantic partners) and non-family members, such as friends and colleagues. A specific distinction was made between NPC and NRL-SC to account for the nuanced circumstances during the pandemic because while it remained possible to engage in real-life social interactions, maintaining a certain distance was mandatory. This allowed for face-to-face meetings, distinct from virtual interactions such as video calls or social media connections, while still prohibiting physical touch. Additionally, participants were asked about their levels of worry, experiences of depressive feelings, and extent of mental fatigue as part of a comprehensive assessment of their mental health. Table A1, in Appendix A, contains the variable names with the question asked and the available answer options.

### 2.3. Statistical Analysis

We estimated a Bayesian Gaussian copula graphical model (BGCGM) and a Bayesian-directed acyclic graph (DAG). A BGCGM is an undirected Bayesian network for mixed data because we included both binary and continuous variables in the network. The nodes represent variables, and the edges show conditional dependence between two variables. The advantage of using Bayesian networks over frequentists is obtaining evidence supporting the inclusion or exclusion of edges. This implies that one can ascertain whether an edge is absent in the network because of genuine conditional independence between two variables or simply because there is insufficient evidence to support the claim of conditional dependence. Edges were included if their Bayes Factor was at least 1, but only those with a factor of at least 10 have strong evidence for inclusion. No prior distribution was used to estimate this network. We used the R package easybgm [13].

For the DAG, arrows pointing to sex and age were blacklisted since these variables cannot be directly influenced by others. This is the only prior distribution that was included in this network. The Hill-Climbing algorithm, a score-based method, was employed to select the optimal network model based on goodness-of-fit measures [14]. We bootstrapped 10,000 samples and applied 50 different random restarts and 100 perturbations to circumvent getting stuck at a local maximum. A network was estimated for each of the 10,000 bootstrapped samples. The final network structure was constructed by averaging the 10,000 estimated networks. The thickness of the arrows represents Bayesian Information Criterion (BIC) values, indicating the significance of an arrow in the network. Variables with lower BIC values make a more substantial contribution to the network structure. Arrows are visible only if the directional probability, the likelihood that an arrow points in a particular direction, exceeds 0.5. All network analyses were performed using R studio version 4.2.2, and the DAG was plotted using R studio version 4.1.2. We used the packages bnlearn [14] and Rgraphviz [15].

T-tests and mixed ANOVAs were performed using SPSS version 28. Data were normally distributed. We estimated Cohen’s d effect sizes and used the cut-offs as proposed by Cohen [16]: *d* = 0.20 as a small effect, *d* = 0.50 as a medium effect, and *d* = 0.80 as a large effect. For the ANOVAs, we estimated partial etas squared and used the cut-offs as proposed by Cohen [16]: η_p_^2^ = 0.0099 as a small effect, η_p_^2^ = 0.0588 as a medium effect, and η_p_^2^ = 0.1379 as a large effect. In all analyses, missing data were handled using pairwise deletion.

## 3. Results

### 3.1. Network Analysis

#### 3.1.1. BGCGM

**Figure 1:**
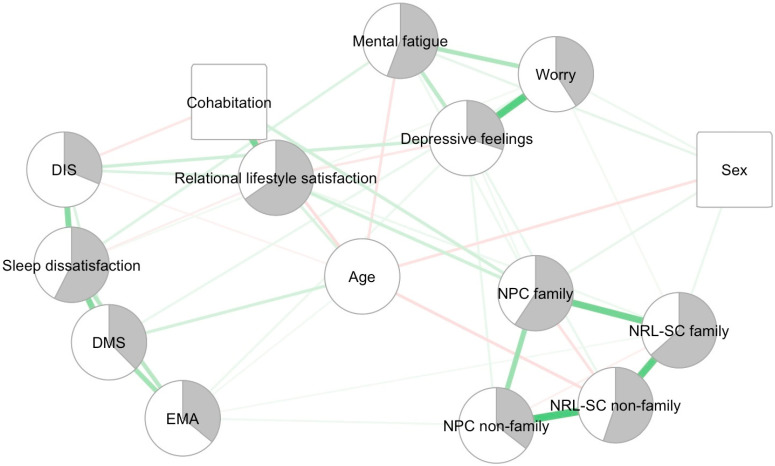
Bayesian Gaussian Copula Graphical Model. Note: Circles represent continuous variables and square binary variables. The grey area in the nodes represents the mean scores on a scale from 1 to 5, a mean score of 3 being represented by half of the node shaded in grey. For relational lifestyle satisfaction, the scale was from 1 to 4. This grey area was not computed for the demographic variables. The weight of the edges is represented by their thickness and color saturation. Positive connections are green, and negative connections are red. An edge between two continuous variables (circles) represents a partial correlation. An edge between two binary variables (squares) or between a binary and a continuous variable represents a regression coefficient. NPC family = need for physical contact with family members; NPC non-family = need for physical contact with non-family members; NRL-SC family = need for real-life social contact with family members; NRL-SC non-family = need for real-life social contact with non-family members; DIS = difficulties initiating sleep; DMS = difficulties maintaining sleep; EMA = early morning awakenings.

**Figure A1:**
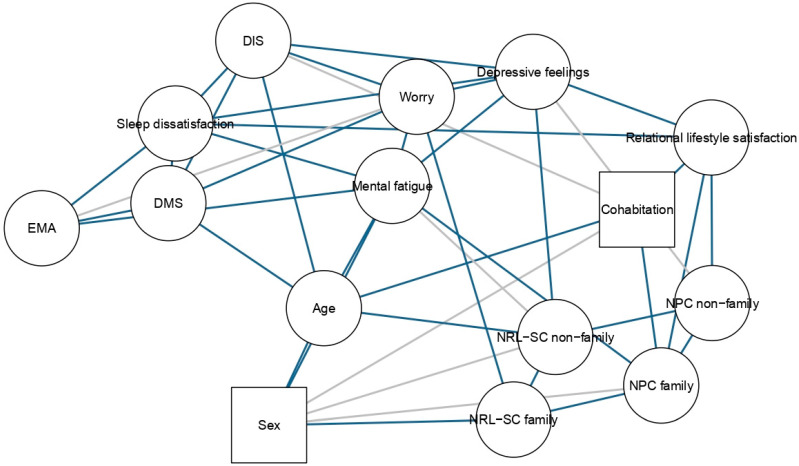
Evidence plot for present edges. Note: Blue represents edges for which there is evidence of presence (BF > 10). Grey represents edges for which there is not enough evidence to include them (1 < BF < 10). NPC family = need for physical contact with family members; NPC non-family = need for physical contact with non-family members; NRL-SC family = need for real-life social contact with family members; NRL-SC non-family = need for real-life social contact with non-family members; DIS = difficulties initiating sleep; DMS = difficulties maintaining sleep; EMA = early morning awakenings.

**Figure A2:**
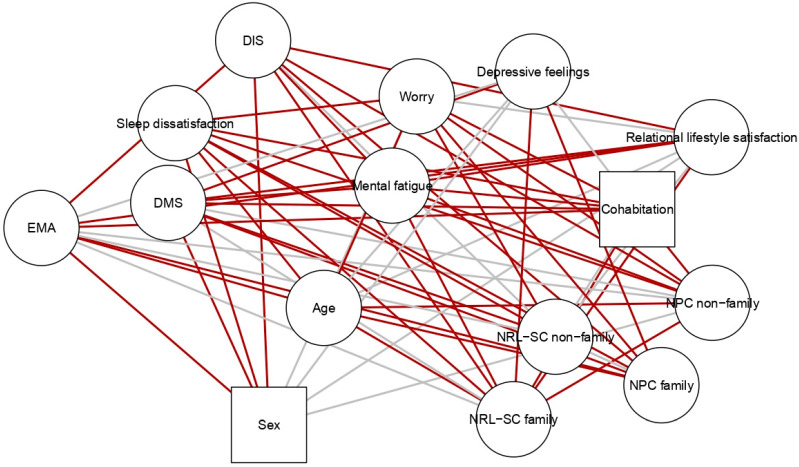
Evidence plot for absent edges. Note: Red represents edges for which there is evidence of absence (BF < 0.1). Grey represents edges for which there is inconclusive evidence to exclude them (0.1 < BF < 1). NPC family = need for physical contact with family members; NPC non-family = need for physical contact with non-family members; NRL-SC family = need for real-life social contact with family members; NRL-SC non-family = need for real-life social contact with non-family members; DIS = difficulties initiating sleep; DMS = difficulties maintaining sleep; EMA = early morning awakenings.

The BGCGM can be seen in Figure 1. The strongest edges in our network are NPC non-family–NRL-SC non-family (0.40), worry–depressive feelings (0.36), NRL-SC family–NRL-SC non-family (0.36), DMS–sleep dissatisfaction (0.35), NPC family–NRL-SC family (0.32), cohabitation–relational lifestyle satisfaction (0.31) and DIS–sleep dissatisfaction (0.30). A table with the edge weights can be consulted in Appendix A, Table A2. Figure A1 and Figure A2 in Appendix A represent the edge evidence plots. All of the edges in the BGCGM have a Bayes Factor of at least 10, indicating strong evidence for inclusion. Several edges have an infinite Bayes Factor.

#### 3.1.2. DAG

**Figure 2:**
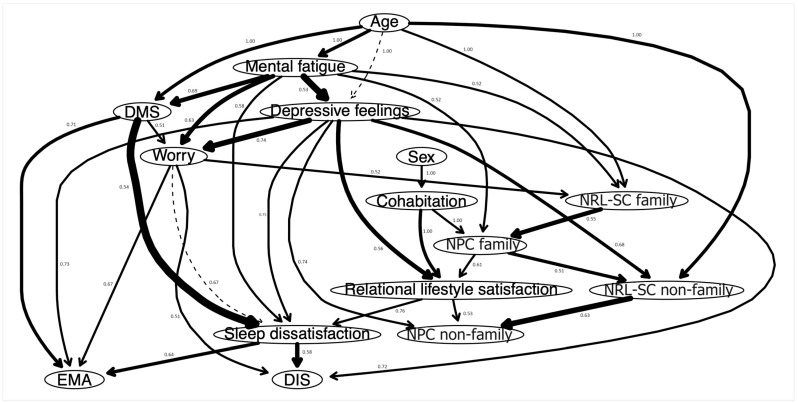
Bayesian-Directed Acyclic Graph. Note: The thickness of an arrow indicates its importance to the overall network model fit. The numbers indicate the likelihood of a given direction. NPC family = need for physical contact with family members; NPC non-family = need for physical contact with non-family members; NRL-SC family = need for real-life social contact with family members; NRL-SC non-family = need for real-life social contact with non-family members; DIS = difficulties initiating sleep; DMS = difficulties maintaining sleep; EMA = early morning awakenings.

The DAG can be seen in Figure 2. The most important arrows to the network structure are DMS–sleep dissatisfaction (BIC: −408.58), mental fatigue–depressive feelings (BIC: −389.66), NRL-SC non-family–NPC non-family (BIC: −354.91), depressive feelings–worry (BIC: −289.18), sleep dissatisfaction–DIS (BIC: −271.69), NRL-SC family–NPC family (BIC: −269.74).

Since we blacklisted arrows going to sex and age, those leaving sex and age obviously have a directional probability of 1. Sex and age are parent nodes, but this could be artificial due to the blacklisting. Cohabitation is the only other variable with departing arrows whose directional probability is 1. Other arrows with strong directional probabilities are relational lifestyle satisfaction to sleep dissatisfaction (0.76), depressive feelings to sleep dissatisfaction (0.75), depressive feelings to worry (0.74), depressive feelings to NPC non-family (0.74), depressive feelings to EMA (0.73), depressive feelings to DIS (0.72) and DMS to EMA (0.71). EMA, DIS, and NPC non-family are child nodes. A table with all the strengths, BIC values, and directional probabilities of the visible arrows can be consulted in Appendix A, Table A3.

### 3.2. Pre- vs. Peri-Lockdown

The reported NPC non-family members and relational lifestyle satisfaction were lower during the second lockdown than before the pandemic. These differences, however, exhibited a small effect size. The reported NRL-SC with family members was higher during the lockdown, but the effect size was negligible. See Table 2 for the results of all *t*-tests.

#### 3.2.1. People Living Alone

**Figure 3:**
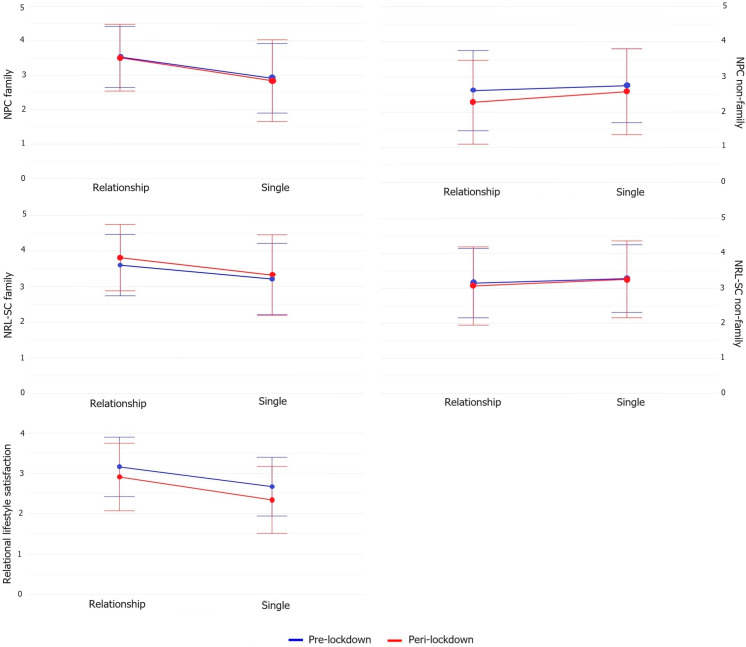
Interaction effects for people not cohabiting.

As apparent from Table 3, no significant interactions were found between pre- vs. peri-lockdown and being in a romantic relationship or not when living alone. Significant main effects of being in a relationship were found for NPC family, NRL-SC family, and relational lifestyle satisfaction. As can be seen in Figure 3, singles showed a lower NPC and NRL-SC with family members compared with those in a relationship. Additionally, those in a relationship (albeit long-distance) were more satisfied with their current relational lifestyle than singles.

#### 3.2.2. People Cohabiting

**Figure 4:**
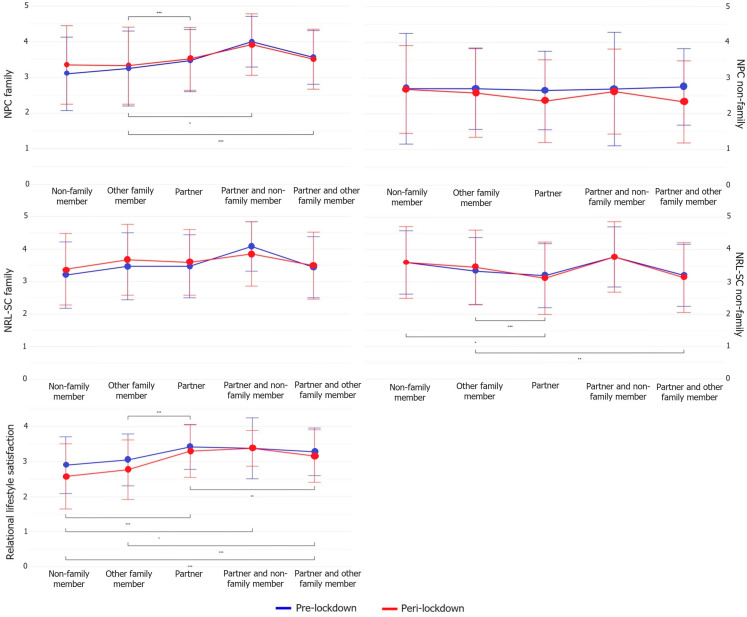
Interaction effects for people cohabiting. Note: * *p* < 0.05, ** *p* < 0.01, *** *p* < 0.001.

These data presented in Table 4 and Figure 4 indicate significant main effects and interactions among cohabiting individuals. Specifically, those residing with their partners, or with both their partners and other family members, reported a reduced NPC with non-family members during the lockdown. This pattern was not observed in other groups. Furthermore, individuals living with their partners or other family members experienced an increased NRL-SC with family members during the lockdown, a trend that did not appear in the other groups. Despite a notable interaction effect, changes in the NPC with family and NRL-SC with non-family were consistent across groups over time. Among those cohabiting with their partner and non-family members, relational lifestyle satisfaction levels remained stable before and during the lockdown. In contrast, the other groups reported a decrease in relational lifestyle satisfaction during the lockdown.

## 4. Discussion

The aim of this study was to explore the interconnection between sleep, mental health, and the need for interpersonal physical and real-life social contact with family and non-family during the second COVID-19 lockdown compared with before, using a Bayesian network analysis.

### 4.1. Network Analysis

Unsurprisingly, people with a greater skin hunger also reported a greater need to see those people in real life (meaning not through a screen). Since physical contact inherently involves seeing someone in real life, a greater need for physical contact logically corresponds to a greater need for real-life social contact. Furthermore, those expressing a greater need for physical and real-life social contact with non-family members also tend to express this need more greatly with family members. This suggests an overall greater requirement for physical and real-life social contact with family than with non-family members. Individuals who express those needs with non-family members tend to have a higher need for physical and real-life social contact with family members, hinting at a general inclination toward increased need for physical and social contact.

Individuals who experience worry are more likely to report difficulties in maintaining sleep, feelings of depression, and mental fatigue. Worrying emerges thus as an important symptom in night-time awakenings, depressive feelings, and mental fatigue and establishes itself as a transdiagnostic factor in the depression–insomnia relationship [17]. Furthermore, people who report worrying, experiencing night-time awakenings, or feeling depressed are more likely to report feeling mentally tired. This means that mental fatigue likely operates as a transdiagnostic factor in the anxiety–depression–insomnia relationship.

Individuals with a greater need for physical and real-life social contact reported poorer mental health (i.e., worry, depression, and mental fatigue), confirming our hypothesis and earlier findings on the importance of interpersonal touch interactions for the quality of life during COVID-19 [8]. Consequently, this means that having a particularly high need for physical and real-life social contact might be a sign of bad mental health. In contrast, people with mental health struggles will not necessarily have a need for touch or social contact. This might be due to their attachment style or negative attitudes towards others and physical contact [9].

Younger individuals reported experiencing fewer night-time awakenings, more mental fatigue, and a greater need to see non-family in real life compared with older individuals. Non-family members, which encompass friends, are more likely to be considered best friends by individuals under 26 years old. Past this age, people tend to designate their partner or a relative as their best friend [18]. Consequently, younger individuals are more inclined to express a greater need for real-life social contact with non-family members.

It is worth noting that despite women being more at risk of suffering from depression, anxiety [19], and insomnia [20], the partial correlations between these variables and sex were small or not present in the network. Moreover, sex was either not or weakly associated with the need for interpersonal touch and social contact.

Despite not blacklisting arrows towards the variable cohabitation in the DAG, all arrows identified (even those not included in the network because of insufficient strength) all had a directional probability of 1 when departing from the cohabitation variable. This implies that in 100% of the cases, cohabitation was solely dependent on sex: people who lived alone were more likely to be men.

People who were more satisfied with their relational lifestyle and who had a greater need for interpersonal touch with family members were more likely to cohabit. The correlation between cohabiting and relational lifestyle satisfaction was one of the strongest in our network. Even though we did not measure relationship satisfaction, previous research by Tai et al. [21] found that couples without intentions of living together report lower relationship satisfaction compared with married couples or couples with intentions of marrying or living together.

Our hypothesis of a negative relationship between relational lifestyle satisfaction, on the one hand, and mental health and sleep complaints, on the other hand, was confirmed by our results. People who were satisfied with their sleep were more satisfied with their relational lifestyle. On the other hand, people who are satisfied with their relational lifestyle are not necessarily satisfied with their sleep. This suggests that being dissatisfied with one’s relational lifestyle might be one of the many factors that impair sleep.

People who were satisfied with their relational lifestyle felt less depressed, tended to cohabitate, and had a greater need for interpersonal touch with family members. Being in a satisfying romantic relationship has been linked with better mental health than being single [22].

### 4.2. Pre- vs. Peri-Lockdown

The need for physical contact with non-family members was lower during the second lockdown compared with before in those cohabiting with their partner and in those cohabiting with their partner and other family members. This contradicts earlier findings of a higher longing for touch linked with the duration of COVID-19 regulations [7]. However, our results can partially align with the results of von Mohr et al. [9] during the first wave of the pandemic, who found that the longer people experienced social distancing, the less they craved physical contact with friends and colleagues, but the more they craved intimate touch (i.e., a kiss, hug, caress from partner or close family). However, contrary to our hypothesis, we did not find a noticeable increase in the need for physical contact with family members during the lockdown compared with before.

Note that our study encompassed data gathered during the second lockdown and not the first lockdown, as in the study by von Mohr et al. [9]. In that regard, another possible explanation might be that having already experienced a lockdown and a loosening of social distancing measures before may have caused the subsequent second lockdown to install a sense of habituation and acceptance of the ongoing abnormal and uncontrollable social circumstances [23], which is reflected in a lower need for interpersonal touch with non-family members. We know that touch deprivation in the long term can lead to a desensitization to interpersonal affective touch [24]. The question then is where the craving [25] for physical contact ends and transits to a sense of habituation and what the mediating factors in that process are. We lack data from the first lockdown to obtain a better picture of this.

However, regarding the need to see family members in real life, this increased in those cohabiting with their partner or with other family members during the lockdown, compared with before. Additionally, those cohabiting with their partner reported a lower need to see non-family members in real life overall compared with those cohabiting with others and non-family members, which can be linked with a decreased need for physical contact with non-family members.

Regarding the need for physical contact with family and the need for real-life social contact with non-family, the groups did not differ in their change over time despite a significant interaction effect. This could be due to the small sample size of the group living with non-family members and the group living with their partner and non-family members. The power might thus be high enough to detect the interaction effect but not to detect the effect of time inside these small groups.

Overall, we found that those living alone who were in a relationship showed a higher need for physical contact and real-life social contact with family members than singles. Similarly, those cohabiting with their partner, or with their partner and other or non-family members, also reported an overall significantly higher need for interpersonal touch with family members compared with those cohabiting with others than their partner. A possible explanation could be that those in a relationship received intimate touch on a regular basis from their partner, causing them to enjoy interpersonal touch from familiar people more [26] and thus seek more physical contact with them [27].

During the second lockdown, people reported lower satisfaction with their relational lifestyle compared with before the lockdown. A study has shown that with regard to life satisfaction (response scale: 0 = “worst possible life”; 10 = “best possible life”), during the lockdowns, singles’ life satisfaction decreased as they had to spend more time alone. In contrast, married couples’ life satisfaction increased as more time was spent with their partner [28]. Relatedly, we found in our study that in those living alone, relational lifestyle satisfaction was higher overall when being in a romantic relationship compared with when being single. In those cohabiting with their partner, or with their partner and other family members or non-family members, this satisfaction was also higher compared with those cohabiting with others than their partner. This is possibly related to previous findings that being in a fulfilling romantic relationship could buffer against COVID-19-related mental health risks [29,30].

Additionally, we found that those cohabiting with their partners and non-family members were the only ones not decreasing relational lifestyle satisfaction before versus during the lockdown. However, the sample size of this group was very small, which could have confounded these results.

### 4.3. Strengths and Limitations

To find significant differences in the *t*-tests with a Cohen’s d of at least 0.20 and a power of 0.90, we needed a sample size of at least 265. To find significant differences in the repeated measures ANOVA with an η_p_^2^ of at least 0.01 and a power of 0.90, we needed a sample size of at least 218 of those non-cohabitating and of at least 305 of those cohabitating. Our sample size was thus large enough. Our study is one of the few that took place during the second COVID-19-related lockdown. By making the questionnaire available in both French and Dutch, we were able to obtain a sample that is more representative of the Belgian population. This approach ensured inclusivity and minimized potential language-related biases that might have arisen if the questionnaire had been accessible in only one language.

It is important to note that the arrows in the DAG indicate directional dependence rather than causal relationships. Directional dependence means that in the relationship x -> y, the presence of the node y more strongly implies the presence of the node x than vice versa [31]. The interpretation of the results regarding the people living with their partner and non-family members was limited due to the small sample size of this subgroup. Furthermore, these pre-lockdown data were collected retrospectively, which led to a potential recall bias. However, as mentioned, these data are part of a larger study wherein we collected data during the first and the second lockdowns. Both questionnaires had many questions in common, including the questions about sleep and mental health, which were used in this study. While we did not collect mail addresses, it is possible that some people replied to both questionnaires. During both lockdowns, participants were asked to recall their pre-pandemic sleep and mental health retrospectively. During the first lockdown, participants were prompted to reflect on a situation that occurred approximately a month prior, a timeframe commonly utilized in questionnaires. During the second lockdown, participants were recalling a situation that occurred more than six months earlier. Despite this temporal discrepancy, our previous analyses indicated that the results regarding pre-pandemic sleep and mental health collected during the second lockdown closely mirrored those obtained during the first lockdown [32]. Consequently, we believe that any potential recall bias was minimal.

This study omitted the measurement of certain concepts such as relationship satisfaction, loneliness, current touch exposure, and longing for touch, leaving gaps in our understanding. Additionally, the broader concept of relational lifestyle satisfaction poses challenges in accurately gauging how individuals interpret it. The interpretation of the results regarding NPC and NRL-SC is limited by not knowing whether that need was fulfilled. It is possible that some people who were not cohabitating with a partner actually had one. However, people cohabiting were not asked whether they were in a relationship. Finally, we lack data from the first lockdown, which limits interpretations.

### 4.4. Implications and Suggestions for Further Research

This paper gives a first impression of the relationship between physical and real-life social contact, mental health, and sleep by means of a network analysis. More research is needed in this field to understand the nature of these relationships better. Note that we obtained data before and during the second lockdown in Belgium but not post-lockdown. Research on post-lockdown effects is valuable in investigating whether the effects found can be recovered in a given time frame. It would also be interesting to investigate possible causal relationships between these variables through temporal networks.

## 5. Conclusions

During the second lockdown, individuals with greater NPC and NRL-SC reported poorer mental health (i.e., worry, depression, and mental fatigue). Younger individuals reported experiencing fewer DMS, more mental fatigue, and a greater NRL-SC with non-family compared with older individuals. Sex was either not or weakly associated with the other variables in our networks despite the well-documented greater prevalence of anxiety, depression, and insomnia among women. Additionally, sleep satisfaction was linked with a higher relational lifestyle satisfaction. Individuals satisfied with their relational lifestyle reported lower levels of depressive feelings and a greater NPC with family members.

NPC with non-family members and relational lifestyle satisfaction was lower during the second lockdown compared with before the pandemic. Overall, individuals in a romantic relationship who are living alone or cohabiting with their partner reported a higher NPC with family members (which includes the partner) and relational lifestyle satisfaction.

## Figures and Tables

**Table 1:** Distribution of with whom the participants cohabitated based on their age categories.

	18–24 Years	25–44 Years	45–64 Years	65+ Years	Total
Lives alone	44	244	194	55	537
Lives only with partner	28	367	257	84	736
Lives with partner and other family members (child, parent, …)	22	407	345	5	779
Lives with other family members (child, parent, …)	314	201	125	3	643
Lives with partner and non-family members (friend, housemate, …)	5	8	2	0	15
Lives with non-family members (friend, housemate, …)	18	32	0	0	50
Total	431	1259	923	147	2760

**Table 2:** *t*-test results.

	Pre-Lockdown (*M*, *SD*)	Peri-Lockdown (*M*, *SD*)	*t*(df)	*p*	*d*
NPC family	3.37 (0.93)	3.39 (1.00)	−0.83 (2094)	0.41	−0.02
NPC non-family	**2.70 (1.10)**	**2.43 (1.19)**	**12.55 (2091)**	**<0.001**	**0.27**
NRL-SC family	**3.43 (0.98)**	**3.55 (1.05)**	**−6.45 (2092)**	**<0.001**	**−0.14**
NRL-SC non-family	3.24 (0.99)	3.23 (1.12)	0.81 (2090)	0.21	0.02
Relational lifestyle satisfaction	**3.18 (0.74)**	**2.98 (0.85)**	**13.82 (2089)**	**<0.001**	**0.30**

Note: Response scale going from 1 to 5, except for relational lifestyle satisfaction going from 1 to 4. Bold indicates significance.

**Table 3:** Mixed ANOVA results for people not cohabiting.

	Main Effect of Being in a Relationship			Interaction Effect of Being in a Relationship and Time		
	*F*(df)	*p*	η_p_^2^	*F*(df)	*p*	η_p_^2^
NPC family	**36.10 (1372)**	**<0.001**	**0.09**	0.26 (1372)	0.61	0.001
NPC non-family	3.45 (1373)	0.06	0.01	1.90 (1373)	0.17	0.005
NRL-SC family	**18.59 (1373)**	**<0.001**	**0.05**	0.80 (1373)	0.37	0.002
NRL-SC non-family	2.24 (1373)	0.14	0.01	0.28 (1373)	0.60	0.001
Relational lifestyle satisfaction	**44.98 (1370)**	**<0.001**	**0.11**	1.26 (1371)	0.26	0.003

Note: Bold indicates significance.

**Table 4:** Mixed ANOVA results for people cohabiting.

	Main Effect Cohabiting			Interaction Effect Cohabiting and Time		
	*F*(df)	*p*	η_p_^2^	*F*(df)	*p*	η_p_^2^
NPC family	**8.33 (4, 1709)**	**<0.001**	**0.02**	**3.67 (4, 1709)**	**0.01**	**0.01**
NPC non-family	1.39 (4, 1705)	0.24	0.003	**7.04 (4, 1705)**	**<0.001**	**0.02**
NRL-SC family	2.25 (4, 1707)	0.06	0.01	**2.72 (4, 1707)**	**0.03**	**0.01**
NRL-SC non-family	**7.63 (4, 1704)**	**<0.001**	**0.02**	**3.30 (4, 1704)**	**0.01**	**0.01**
Relational lifestyle satisfaction	**36.07 (4, 1707)**	**<0.001**	**0.08**	**6.31 (4, 1707)**	**<0.001**	**0.02**

Note: Bold indicates significance.

## Data Availability

The dataset analyzed during the current study is available at the Open Science Framework, https://osf.io/tv2dw/, accessed on 2 February 2024.

## Appendix A

**Table A1:** Items and answer options.

Variable Name	Item	Answer Options
Cohabitation		I live alone/I do not live alone
Relational lifestyle satisfaction	Since the second lockdown, I am … about my current relational lifestyle (married, in a romantic relationship, …).	Very unsatisfied/unsatisfied/satisfied/very satisfied
Need for physical contact with family	Since the second/before (the) lockdown, I need(ed) physical contact or hugs with my family and/or partner.	Not at all/slightly/moderately/a lot/extremely
Need for real-life social contact with family	Since the second/before (the) lockdown, I need(ed) to see my family and/or partner outside of virtual contacts (video calls, social media, …).	Not at all/slightly/moderately/a lot/extremely
Need for physical contact with non-family	Since the second/before (the) lockdown, I need(ed) physical contact or hugs with non-family members (friends, colleagues, …).	Not at all/slightly/moderately/a lot/extremely
Need for real-life social contact with non-family	Since the second/before (the) lockdown, I need(ed) to see non-family members (friends, colleagues, …) outside of virtual contacts (video calls, social media, …).	Not at all/slightly/moderately/a lot/extremely
DIS	Difficulty falling asleep	None/mild/moderate/severe/very severe
DMS	Difficulty staying asleep	None/mild/moderate/severe/very severe
EMA	Problem waking up too early in the morning	None/mild/moderate/severe/very severe
Sleep dissatisfaction	How satisfied/dissatisfied are you with your current sleep pattern?	Very satisfied/satisfied/neutral/dissatisfied/very dissatisfied
Mental fatigue	Since/before the lockdown, I need(ed) to get some mental rest	Not at all/slightly/moderately/a lot/extremely
Worry	Since/before the lockdown, I feel/felt worried	Not at all/slightly/moderately/a lot/extremely
Depressive feelings	Since/before the lockdown, I feel/felt depressed	Not at all/slightly/moderately/a lot/extremely

**Table A2:** Edge weights of the BGCGM.

	X1	X2	X3	X4	X5	X6	X7	X8	X9	X10	X11	X12	X13	X14	X15
X1															
X2	−0.134														
X3	0.042	−0.130													
X4	0.014	0.010	0.308												
X5	0.044	−0.000	0.146	0.092											
X6	−0.015	−0.000	−0.000	−0.128	0.210										
X7	0.104	−0.002	0.000	0.000	0.320	−0.001									
X8	−0.068	−0.016	−0.005	−0.007	−0.024	0.397	0.356								
X9	0.002	−0.054	−0.065	0.000	0.000	0.000	0.000	0.006							
X10	0.000	0.166	0.000	0.000	0.000	0.003	−0.004	0.000	0.114						
X11	−0.003	0.002	0.000	−0.002	0.000	0.007	0.003	0.004	0.000	0.220					
X12	−0.000	0.000	0.000	−0.071	0.000	0.000	0.000	−0.000	0.297	0.346	0.219				
X13	0.090	−0.143	0.000	−0.000	0.083	0.000	0.000	0.032	0.000	0.002	0.068	0.146			
X14	0.013	−0.000	0.000	0.003	0.000	0.000	0.080	0.000	0.074	0.118	0.054	−0.000	0.273		
X15	−0.001	−0.019	−0.003	−0.186	0.000	0.056	0.000	0.069	0.107	0.000	0.014	0.065	0.217	0.359	

Note: X1 = sex; X2 = age; X3 = cohabitation; X4 = relational lifestyle satisfaction; X5 = need for physical contact with family; X6 = need for physical contact with non-family; X7 = need for real-life social contact with family; X8 = need for real-life social contact with non-family; X9 = difficulties initiating sleep; X10 = difficulties maintaining sleep; X11 = early morning awakenings; X12 = sleep dissatisfaction; X13 = mental fatigue; X14 = worry; X15 = depressive feelings.

**Table A3:** Strength, BIC values, and directional probabilities.

From	To	Strength	BIC Value	Directional Probability
Sex	Cohabitation	0.55	−0.39	1.00
Age	NRL-SC family	0.68	−24.41	1.00
Age	NRL-SC non-family	1.00	−44.87	1.00
Age	DMS	1.00	−47.65	1.00
Age	Mental fatigue	1.00	−59.93	1.00
Age	Depressive feelings	0.55	2.47	1.00
Cohabitation	Relational lifestyle satisfaction	1.00	−72.44	1.00
Cohabitation	NPC family	1.00	−33.56	1.00
Relational lifestyle satisfaction	NPC non-family	0.99	−6.09	0.53
Relational lifestyle satisfaction	Sleep dissatisfaction	0.94	−6.31	0.76
NPC family	Relational lifestyle satisfaction	1.00	−28.28	0.61
NPC family	NRL-SC non-family	0.56	−49.29	0.51
NRL-SC family	NPC family	1.00	−269.74	0.55
NRL-SC non-family	NPC non-family	1.00	−354.91	0.63
DMS	EMA	1.00	−95.65	0.71
DMS	Sleep dissatisfaction	1.00	−408.58	0.54
DMS	Worry	0.99	−28.36	0.51
Sleep dissatisfaction	DIS	1.00	−271.69	0.58
Sleep dissatisfaction	EMA	1.00	−66.32	0.64
Mental fatigue	NPC family	0.89	−7.36	0.52
Mental fatigue	NRL-SC family	0.52	−11.43	0.52
Mental fatigue	DMS	0.59	−169.51	0.69
Mental fatigue	Sleep dissatisfaction	1.00	−32.25	0.58
Mental fatigue	Worry	1.00	−113.70	0.63
Mental fatigue	Depressive feelings	1.00	−389.66	0.53
Worry	NRL-SC family	0.88	−11.68	0.52
Worry	DIS	0.55	−1.19	0.51
Worry	EMA	0.61	−0.18	0.67
Worry	Sleep dissatisfaction	0.53	0.58	0.67
Depressive feelings	Relational lifestyle satisfaction	1.00	−164.29	0.56
Depressive feelings	NPC non-family	0.88	−9.09	0.74
Depressive feelings	NRL-SC non-family	0.88	−61.01	0.68
Depressive feelings	DIS	1.00	−22.76	0.72
Depressive feelings	EMA	0.77	−1.83	0.73
Depressive feelings	Sleep dissatisfaction	0.64	−3.25	0.75
Depressive feelings	Worry	1.00	−289.18	0.74

Note: NPC family = need for physical contact with family members; NPC non-family = need for physical contact with non-family members; NRL-SC family = need for real-life social contact with family members; NRL-SC non-family = need for real-life social contact with non-family members; DIS = difficulties initiating sleep; DMS = difficulties maintaining sleep; EMA = early morning awakenings.
